# Circular RNA‐mediated ceRNA network was identified in human lung adenocarcinoma by high‐throughput sequencing

**DOI:** 10.1111/1759-7714.14884

**Published:** 2023-05-03

**Authors:** Yongyang Chen, Xiaoling Han, Xiaobi Huang, Honglian Zhou, Hui Yu, Lihui Wang, Zijian Liu, Baiyang Liu, Jian Huang, Yinghuan Xiong, Jian Huang, Yang Shao, Dongqin Zhu, Zhu Liang, Zhixiong Yang, Wenmei Su

**Affiliations:** ^1^ Department of Pulmonary Oncology Affiliated Hospital of Guangdong Medical University Zhanjiang China; ^2^ Second Faculty of Clinical Medicine Guangdong Medical University Zhanjiang China; ^3^ Key Laboratory of Longevity and Aging‐Related Diseases of Chinese Ministry of Education, Center for Translational Medicine & School of Preclinical Medicine Guangxi Medical University Nanning China; ^4^ Department of Thoracic Surgery Maoming People's Hospital Maoming China; ^5^ Technical Department Nanjing Geneseeq Technology Inc. Nanjing China; ^6^ Guangdong Provincial Key Laboratory of Autophagy and Major Chronic Non‐communicable Diseases Affiliated Hospital of Guangdong Medical University Zhanjiang China

**Keywords:** circular RNAs, high‐throughput sequencing, lung adenocarcinoma

## Abstract

**Objectives:**

Aberrantly expressed circular RNAs (circRNAs) have been detected in many types of tumors. Hence, they are currently investigated as candidate biomarkers for diagnostic and potential targets for therapy in cancers. The objective of this study was to assess the expression profile of circRNA in lung adenocarcinoma (LUAD).

**Methods:**

This study included 14 pairs of postoperative lung adenocarcinoma specimens, including cancer tissues and matched adjacent tissues. Second‐generation sequencing was applied to the specimens to determine the circRNA expression in them among the 5242 distinct circRNAs detected.

**Results:**

We identified a total of 18 significantly dysregulated circRNAs in the LUAD tissues: upregulation in four and downregulation in 14. ROC (The receiver operating characteristic curve) further suggested that hsa_circ_0120106, has_circ_0007342, has_circ_0005937, and circRNA_0000826 could potentially be used as biomarkers in the diagnosis of LUAD. Furthermore, study of the circRNA–microRNA (miRNA)–messenger RNA (mRNA) revealed interactions between the 18 dysregulated circRNA and several cancer‐related miRNAs. Finally, a further Kyoto Encyclopedia of Genes and Genomes analysis showed that the cell cycle phase transition, p53 signaling pathway, AMP‐activated protein kinase (AMPK) relative signaling pathway, and so on were key putative pathways in the process of LUAD.

**Conclusions:**

These findings demonstrated the correlation between abnormality in circRNA expression and LUAD, which lays the foundation of making CircRNAs candidate biomarkers in the diagnosis of LUAD.

## Graphical abstract

CeRNA regulating network was identified from 14 pairs of postoperative lung adenocarcinoma by RNA‐seq analysis

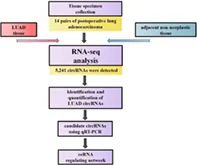

## INTRODUCTION

Lung cancer has the highest morbidity and mortality among malignant tumors.^1^ About 85% of all lung cancer cases are non‐small‐cell lung cancer (NSCLC), whose 5‐year survival rate is only 16%.^2^, ^3^ Early NSCLC mainly depends on surgical treatment, but at present most NSCLC patients are in the middle and late stages when they are first diagnosed, which affects the treatment and prognosis of patients with lung cancer because of the failure to diagnose lung cancer in time. At present, the clinical diagnosis and treatment of lung cancer have been improving. Clarifying the molecular mechanism of NSCLC would contribute to timely, reliable diagnosis by proposing new biomarkers and improve prognosis by locating new therapeutic targets.

Circular RNA (circRNAs) is the third largest family of noncoding RNA after microRNAs (miRNAs) and long non‐coding RNA (lncRNAs). They are characterized by a noncovalent closed‐loop structure at the 3′ and 5′ ends, which leads to significant tolerance to exonuclease^4^ and sequence conservation. In the past, circRNAs have been considered to be a by‐product of low abundance splicing or splicing errors.^5^ The rapid progress in second‐generation sequencing and bioinformatics technology has seen the indispensable role played by circRNAs in human gene expression being more and more discovered.^6^ Recent evidence suggests that circRNAs participate in many normal physiological processes and their abnormalities contribute to the pathogenesis of various diseases, including lung cancer.^7^ For example, in the diagnosis of NSCLC patients with the echinoderm microtubule‐associated protein‐like 4 (EML4)‐gene‐anaplastic lymphoma kinase (ALK) fusion gene, the circRNA F‐CircEA expressed from this fusion gene can be used as a promising fluid biopsy biomarker.^8^ In addition, circRNAs take part in a variety of biological processes via regulation of gene expression. This is accomplished through interactions with miRNAs and other competing endogenous RNAs.^9^, ^10^ As a sponge of miRNA, they regulate gene transcription, regulate RNA‐binding proteins, and participate in protein translation.^11^ There is increasing evidence that the abnormal expression of circRNA may contribute to the occurrence of different cancers, such as lung cancer,^12^, ^13^ esophageal squamous cell carcinoma,^14^ hepatocellular carcinoma,^15^ and colorectal cancer.^16^ It can also potentially provide insights for the development of new targeted therapies. Up to now, the expression profile and possible role of circRNAs in lung adenocarcinoma (LUAD) have been unclear to a large extent, therefore the objective of this study was to identify circRNAs with the potential to act as new biomarkers in the diagnosis and prediction of NSCLC.

To our knowledge, this study collected the largest number of clinical specimens for the detection of the expression of circRNA in lung cancer and adjacent tissues. In this study, we obtained the differential expression of circRNAs in tissue samples of LUAD tissues and nontumor tissues through high‐throughput sequencing and bioinformatics analysis. We established that chr2|43 973 004|43 992 865|+hsa_circ_0120106, has_circ_0007342, has_circ_0005937, and circRNA_0000826 are promising biomarkers for LUAD.

## METHODS

### Tissue specimen collection

Samples were collected from patients who were diagnosed with LUAD and were treated with radical resection in the department of thoracic surgery at the Affiliated Hospital of Guangdong Medical University from October 2019 to December 2019. Before admission, none of these patients had been treated with any other cancer therapy. From each patient, a pair of specimens was collected, a LUAD tissue specimen and one from an adjacent non‐neoplastic tissue. The specimens were fresh frozen and stored in liquid N_2_ immediately on collection. Among the 18 pairs of specimens, four pairs were excluded because of significant RNA degradation that meant they did not meet the requirements. The remaining 14 pairs of specimens then underwent second‐generation sequencing. To verify the result, another 10 pairs of specimens were tested by real‐time qPCR. All the LUAD specimens were subject to histopathological examination in parallel with the aforementioned molecular biological study (Cell proliferation, transwell migration and invasion, Colony formation, cell cycle and so on). This study was approved by the Ethics Committee of the Affiliated Hospital of Guangdong Medical University, and each participant signed an informed consent form.

### RNA‐seq analysis

According to the characteristics of the biological sample, we first extracted all the transcribed RNA of the biological sample, and then selected an appropriate method for sample quality evaluation. Qualified samples were subjected to ribosomal removal, reverse transcription, library construction, and sequencing. The RNA was interrupted and the first strand was synthesized by reverse transcription with random primers. In the second strand synthesis, deoxyuridine 5’‐triphosphate (dUTP) was used instead of deoxythymidine triphosphate (dTTP). After the end repair, Adenine was added to connect the connector. The second strand containing U was then removed under the action of the USER enzyme, leaving only one strand, and PCR amplification was performed on the first strand. The RNA‐seq with chain‐specific libraries has such data characteristics after on‐machine sequencing: read 1 measuring the antisense strand and read 2 measuring the sense strand. After the library was qualified, illumina MiSeq/NextSeq/HiSeq sequencing was performed after pooling the different libraries according to the requirements of effective concentration and target data volume.

### Identification and quantification of LUAD circRNAs

A high‐throughput sequencer (Illumina HiSeq 4000) gave the paired‐end reads, quality controlled by Q30. The Spliced Transcripts Alignment to a Reference (STAR) software aligned the reads to the reference genome/transcriptome. CircRNAs were identified and recorded with DCC software. The identified circRNAs were then annotated based entries retrieved from databases (CircBase and circ2Trait). Differentially expressed circRNAs were identified by Student's *t*‐test between two groups.

### Validation of candidate circRNAs using qRT‐PCR

The circRNA extracted from the LUAD and matching normal tissues (*n* = 14 pairs) were used as templates to synthesize Complementary DNA (cDNA) with a reverse transcriptase according to the manufacture’s instructions. circRNA expression was measured using qPCR (SYBR Green PCR Master Mix; Applied Biosystems). An ABI 7300 Real Time PCR System (Applied Biosystems) was used to perform the PCR following the protocol 1× 2‐min cycle at 50°C, 1× 10‐min cycle at 95°C, 40× 15‐s cycle at 95°C, and 1 min at 60°C. β‐actin served as a control. PCR was conducted three times on each sample.

### Prediction of circRNA‐miRNA interactions

In this study, miRanda^17^ was used to predict the possibility of circRNA binding to known miRNAs. The miRanda algorithm is divided into two steps: (i) a dynamic programming local comparison was performed between miRNA and circRNA, which gives a score based on the complementarity of the sequence, (ii) take out the high‐scoring comparison results to estimate miRNA and circRNA forms the thermodynamic stability of RNA double‐strands. The smaller the free energy, the more stable the combination of the both, therefore sequences with free energy lower than a certain threshold can become miRNA target sequences. Using miRanda software, this process will give the possible binding miRNAs and the number of miRNAs originating from the corresponding species in miRBase^18^ for the circRNA in the circBase annotation.

### Construction of the miRNA–messenger RNA (mRNA) interaction network

Following these investigations, potential circRNA binding miRNAs were isolated, and their potential target genes were predicted by miRWalk 2.0. MiRWalk, miRDB, and the Targetscan database were chosen to predict miRNA target mRNAs. The target genes validated by experiments were downloaded from validated target modules. The miRNA–target gene interaction pairs were selected if they were predicted in two or more databases and validated by experiments. The miRNA–target genes network was visualized using Cytoscape (Version 3.2.1). Gene ontology (GO) and Kyoto Encyclopedia of Genes and Genomes (KEGG) pathway enrichment analyses for target genes of miRNAs were performed using Metascape.

### Construction of the Competing endogenous RNA (ceRNA) regulating network

The circRNA–miRNA and miRNA–mRNA networks were combined to obtain the circRNA–miRNA–target genes network as a ceRNA network.

### Statistical analysis

Relative circRNA expression was calculated using the 2^‐(∆∆Ct) method. Student's *t*‐test at the 5% level of significance was used to determine the significance for differences in circRNA expression. Statistical analysis was carried out using SPSS and Microsoft Excel.

## RESULTS

### Identification of differentially expressed circRNA

**FIGURE 1:**
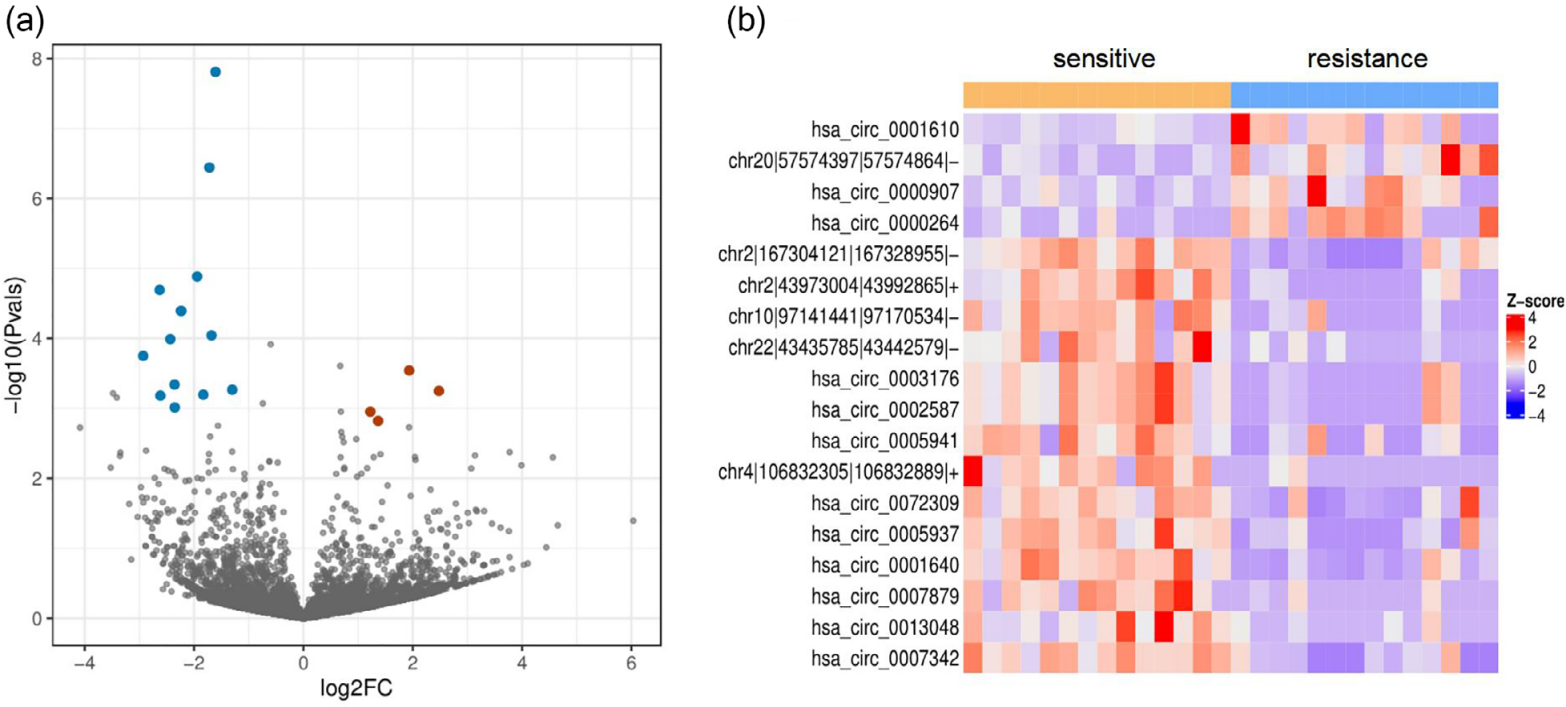
(a) Volcano plots showing differential expression of circRNAs between cancerous and normal tissue. Differentially expressed circRNAs with fold change ≥2.0 (log2 scaled) and *p* < 0.05 (−log10 scaled) are represented by red dots. (b) Hierarchical clustering analysis of the 18 significantly dysregulated circRNAs

To analyze the expression of circRNA in LUAD and explore the potential role of new circRNA biomarkers in the disease diagnosis and prognosis, we performed second‐generation sequencing on our specimens. A total of 5241 circRNAs were detected (Supporting Information Table S1). Cancer tissue exhibits significant differential expression of circRNAs compared to adjacent noncancerous tissue. (Figure 1a,b). Eighteen candidate circRNAs were picked out via sequencing and subsequent overlap analysis whose differential expression was statistically significant. Comparing to adjacent noncancerous tissues, LUAD tissues show upregulation of four of these circRNAs and downregulation of the other 14 (Supporting Information Table S2). The multiple variation of circRNAs expressed between the two groups with fold‐changes was greater than 1.0, and the *p* adjust was less than 0.05. The expression levels of hsa_circ_0000264, chr20|57 574 397|57 574 864|‐, hsa_circ_0000907, and hsa_circ_0001610 in LUAD patients were upregulated, while the expression levels of chr4|106 832 305|106 832 889|+, hsa_circ_0120106, hsa_circ_0007879, chr10|97 141 441|97 170 534|‐, hsa_circ_0013048, chr22|43 435 785|43 442 579|‐, hsa_circ_0003176, hsa_circ_0002587, has_circ_0005937, hsa_circ_0005941, chr2|167 304 121|167 328 955|‐, hsa_circ_0072309, hsa_circ_0001640, and hsa_circ_0007342 in LUAD patients were downregulated. Since hsa_circ_0003176 and hsa_circ_0002587 have the same gene name and gene ID, subsequent analysis will classify these as circRNAs.

### Potential biomarker of circRNA for NSCLC diagnosis

**FIGURE 2:**
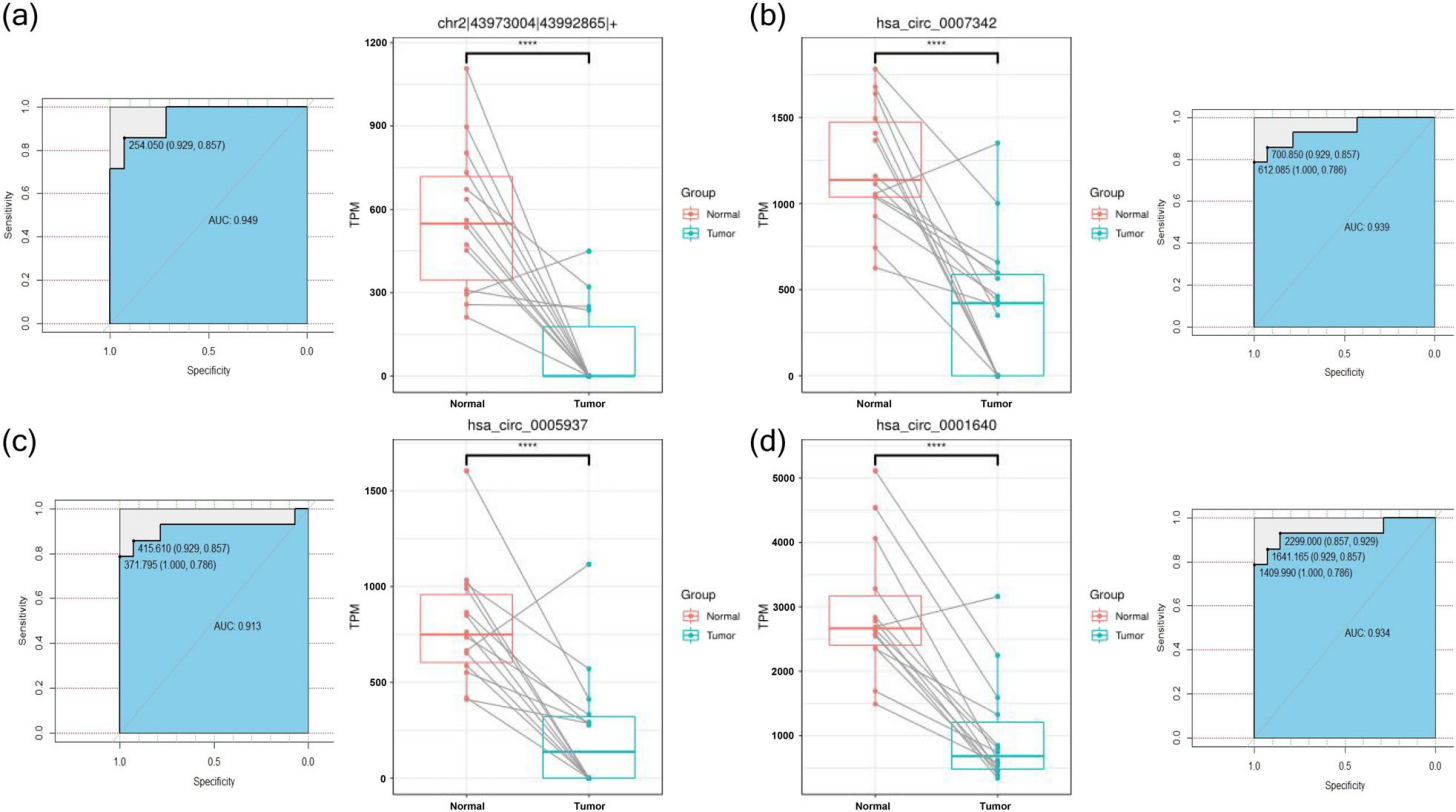
The higher expression levels of (a) hsa_circ_0120106, (b) has_circ_0007342, (c) has_circ_0005937, and (d) circRNA_0001640 in NSCLC cases than that in healthy controls. Data are shown as mean ± SD, ***p < 0.001. ROC curve analysis of differentially expressed circRNAs. AUC values are given on the graphs

CircRNAs have great potential to be used as diagnosis markers for NSCLC. We applied a logistic regression model with LASSO（Least absolute shrinkage and selection operator) regularization to test this idea. Using only seven out of the 18 differentially expressed genes, we were able to achieve 100% prediction accuracy with AUC（Area Under roc Curve） = 1. The seven genes included one gene upregulated in a tumor and the others all downregulated in matched normal samples. Furthermore, we examined the potential of using individual genes as diagnosis markers by conducting receiver operating characteristic (ROC) curve analysis for single genes. Four of the 18 individual genes exhibited AUC > 0.9, suggesting the potential to use single genes for diagnosis. In the ROC curve (Figure 2), the AUC to discriminate LUAD from normal tissues was 0.949 for chr2|43 973 004|43 992 865|+, 0.939 for hsa_circ_0007342, 0.934 for hsa_circ_0001640, and 0.913 for hsa_circ_0005937, and all of them were downregulated in LUAD tissues. These results confirm the potential of these circRNAs as biomarkers in the diagnosis and prediction of LUAD.

### Verification of differentially expressed circRNAs

**FIGURE 3:**
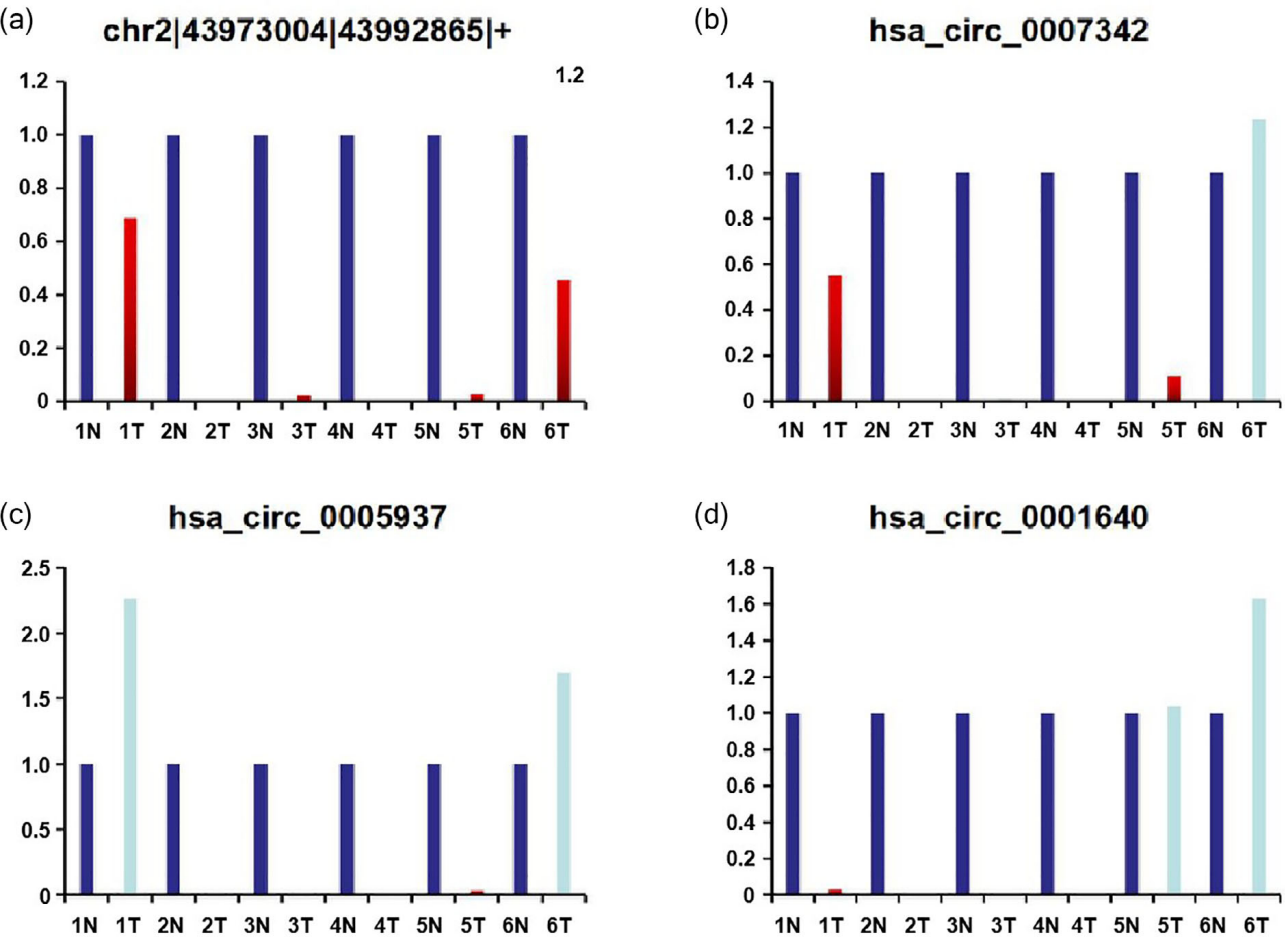
Relative expression of hsa_circ_0120106, has_circ_0007342, has_circ_0005937, and circRNA_0001640 in another independent cohort of NSCLC patient samples

Considering that the ROC has significant specificity and is clinically relevant, we focused on the circRNAs of AUCs higher than 0.9. From the list in Supporting Information Table S2, we first selected the four circRNAs hsa_circ_0120106, has_circ_0007342, has_circ_0005937, and circRNA_0001640 whose differential expression between cancerous and noncancerous tissues is most significant. To verify the circRNA‐seq results of these four circRNAs, qRT‐PCR was performed in the tissue samples from six LUAD and adjacent normal tissues. Figure 3 shows significant downregulations of circRNAs hsa_circ_0120106, has_circ_0007342, has_circ_0005937, and circRNA_0001640 in LUAD patients. This further supports our results obtained from sequencing.

### Functional enrichment analyses for differentially expressed circRNAs

**FIGURE 4:**
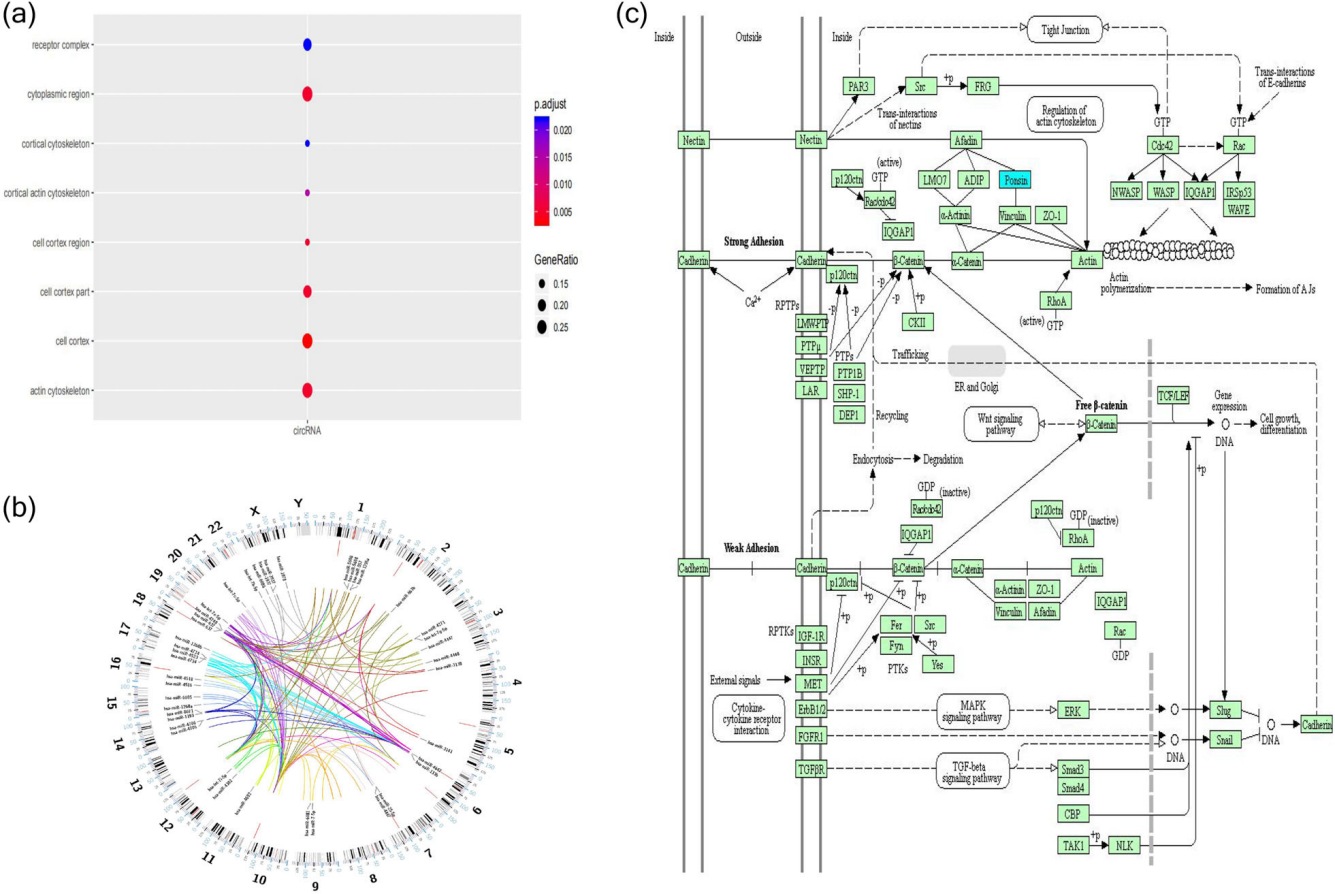
(a) Bulb map of KEGG analysis for 18 circRNA target gene‐related significant enriched signaling pathways. The *y* axis shows the name of statistics pathway enrichment. The area of each node represented the number of enriched differential genes. The *p* values are indicated by color changes from red to purple. (b) Circos plots showing all circRNAs from cell. (c) Enrichment analysis of target genes of ceRNA. Top 100 enriched GO terms in target genes regulated by ceRNA

In addition, Gene Oncology (GO) and Kyoto Encyclopedia of Genes and Genomes (KEGG) and genome of path analysis showed that target genes that were related to this circRNA signature participated in various biological processes, including receptor complex, cytoplasmic region, and cortical cytoskeleton cancer‐related pathways (Figure 4a,b). The circRNAs found in this study to be dysregulation in LUAD tissues intimately involves several key signaling pathways, such as the Wnt signaling pathway, the MARK signaling pathway, and the Transforming growth factor (TGF)‐beta signaling pathway (Figure 4c). As this was a single‐center study, the sample size was relatively small and larger studies are needed to evaluate the role of statistical power of circRNAs in NSCLC.

### Prediction of circRNA‐miRNA interactions

**FIGURE 5:**
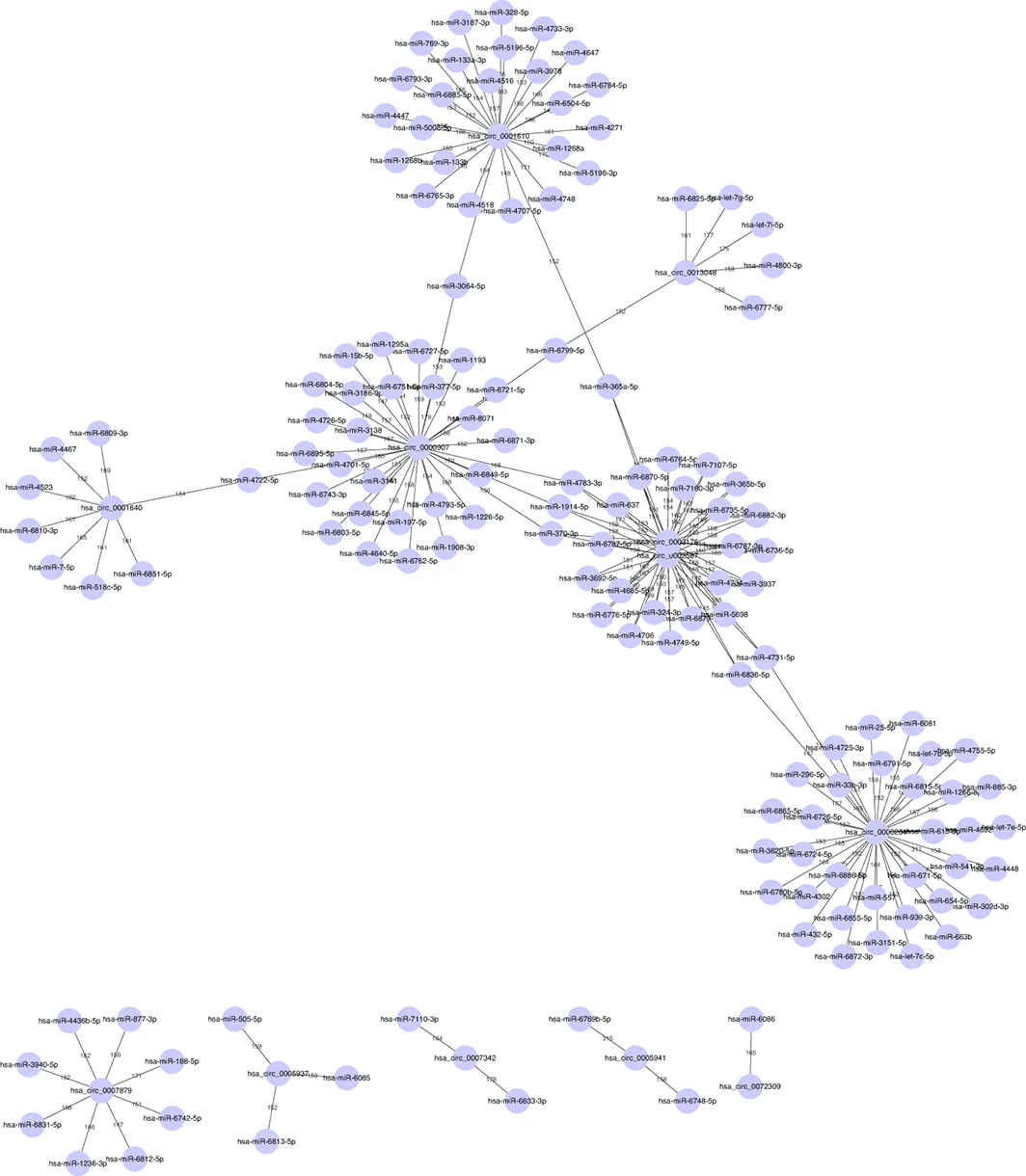
circRNA and miRNA binding network

Previous works have established that circRNA regulates gene expression via a variety of mechanisms, such as inhibiting miRNAs by absorbing them. We identified and ranked miRNAs that bind to LUAD‐related circRNAs. Circos plots drawing the predicted binding relationship between circRNA and miRNA are shown in Figure 5 (Supporting Information Table S2).

### Construction of the CircRNA–miRNA–mRNA ceRNA network

**FIGURE 6:**
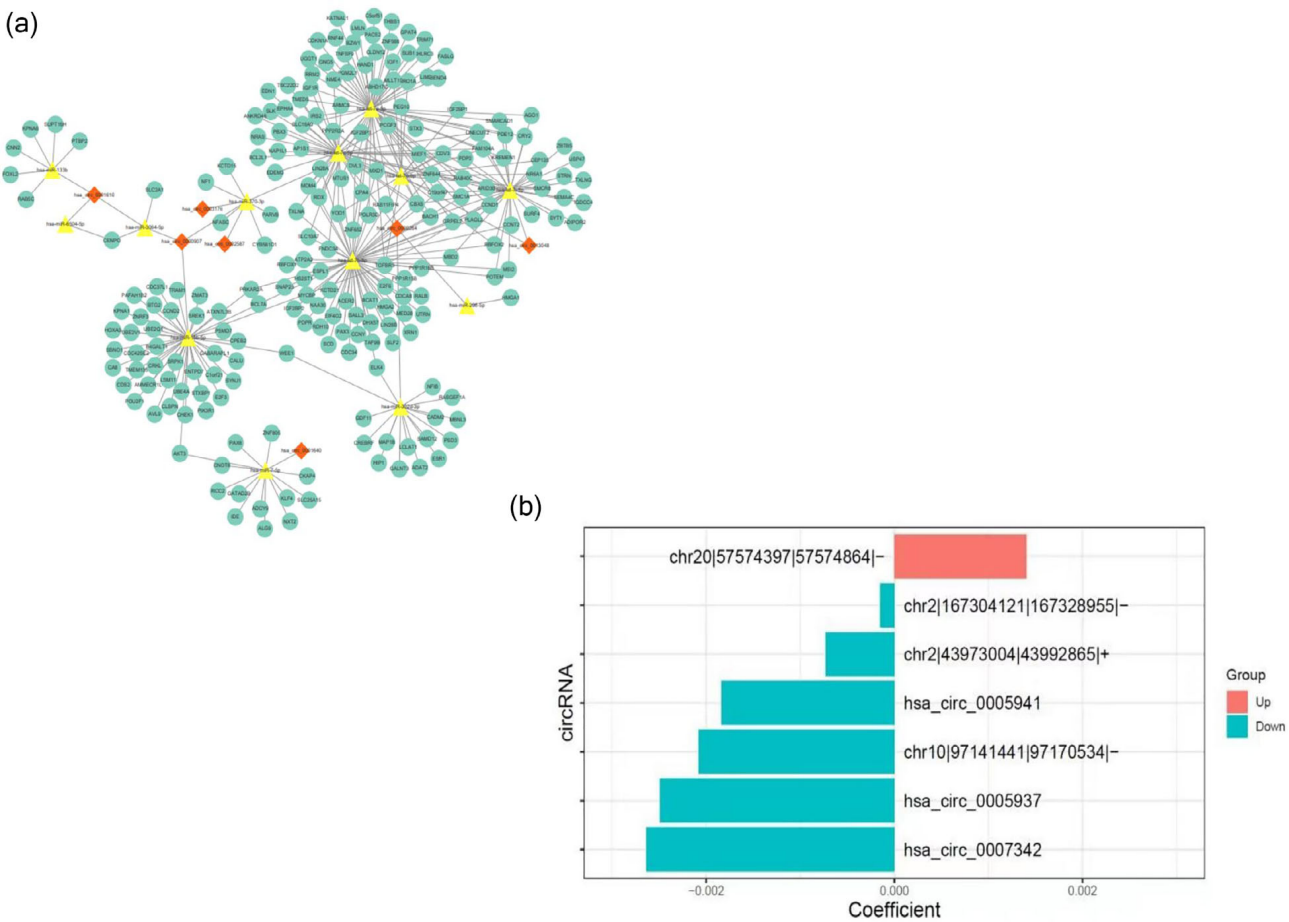
(a) Competing endogenous RNA (ceRNA) regulating networks. (b) Coefficients of circRNAs from a LASSO logistic model for NSCLC diagnosis prediction

According to ceRNA theory, based on the miRNA that binds to circular RNA, miRwalk^19^ is used to predict miRNA target genes. The predicted target genes were screened under the following conditions: (i) there are results in the other two prediction websites and (ii) the published papers confirm that there is a binding between miRNA and target genes. A total of 217 target genes were screened (Figure 6a and Table S3). Metascape analyzed the GO, KEGG, and protein interaction network of candidate proteins. Figure 6a shows the GO and KEGG cluster analysis results of 217 target genes. The results suggest that target genes are closely related to cell cycle, p53 signaling pathway, AMP‐activated protein kinase (AMPK) signaling pathway, other signalling pathways that affect cell function (*p* < 0.05) (Supporting Information Table S4). By using a logistic model with LASSO regularization, we selected seven circRNAs that combination achieved 100% prediction accuracy. Figure 6b shows the coefficients and the direction of the seven genes.

## DISCUSSION

The mechanism and role of circRNAs in cancer progression are not clear.^20^ Emerging evidence has found that circRNAs play an important role in multiple pathological and physiological functions. The available evidence shows that circRNAs are associated with proliferation,^21^ cell cycle,^22^ aoptosis,^23^ and autophagy.^24^ Transcriptional regulation underlies the complexity of LUAD, and its misregulation can contribute to disease.^6^ Indeed, several studies focused on the linear transcriptome have identified co‐expression networks and changes in splicing associated with LUAD status.^25^ Here, we provide insight into the LUAD‐associated circular transcriptome.

We identified dysregulated circRNAs in 14 candidate circRNAs in lung cancer and adjacent tissues for the first time. It has been reported that circRNAs are often downregulated in cancer.^26^ We found that 17 significantly circRNAs from the results of a RNA‐seq experiment, 4 upregulated and 13 downregulated. The downregulated circRNAs hsa_circ_0120106, has_circ_0007342, has_circ_0005937, and circRNA_0001640 had AUCs higher than 0.9 in the ROC curve. It is reported in the literature that hsa_circ_0120106 can recruit TGFB1I1, and TGFB1I1 is involved in cell adhesion and regulation of androgen receptor activity,^27^ thus it plays an important role in cell proliferation and migration. Hsa_circ_0001640 is involved in protein binding and chemical transmission between synapses. Hsa_circ_0005937 is involved in cell adhesion, the RAS signaling pathway, megakaryocyte development, and platelet production. So far, the functions of has‐circ‐000742 remain unclear. Four differentially expressed circRNAs were selected for RNA‐seq data validation by RT‐qPCR. That means these four circRNAs as the potential biomarkers in the diagnosis and prediction of LUAD.

Supporting Information Table S2 shows that 11 circRNAs possessed miRNA binding sites in the ceRNA network, including three upregulated circRNAs (hsa_circ_0000264, hsa_circ_0000907, and hsa_circ_0001610) and eight downregulated circRNAs (hsa_circ_0001640, hsa_circ_0002587, hsa_circ_0013048, hsa_circ_0007879, hsa_circ_0005937, hsa_circ_0072309, hsa_circ_0005941, and hsa_circ_0007342), with most not being reported in circBase.

In the ceRNA network, we enriched many functions, and the mRNA pathways were associated with LUAD in GO and KEGG pathway analyses (Supporting Information Table S4). In the results of our analysis, we found that some genes are targeted by circRNAs that are upregulated in LUAD, such as RRM2, insulin‐like growth factor 2 mRNA binding protein 3 (IGF2BP3), and HMGA. It has been reported that the ribonucleotide reductase subunit M2 (RRM2) is closely related to malignant tumor biological behavior and metastatic potential. With the knockdown of the expression level of RRM2, the activity of ribonucleotide reductase, the growth of cancer cells, and tumor metastasis were downregulated. Meanwhile, tumor cell apoptosis increased. According to various studies, RRM2 can also be used as a target for antilung cancer drugs.^28^ IGF2BP3 is a member of the IGF2BPs family, which are consider having a marked impact on cancer occurrence and development. Guo et al. found that IGF2BP3 was abnormally highly expressed in LUAD and this could lead to a weaker prognosis (*p* < 0.05). IGF2BP3 may therefore be an oncogene and potential prognostic biomarker of LUAD.^29^ In the study by Stewart et al., high mobility group A1 (HMGA1) was implicated in tumorigenesis. HMGA1 has recently been described as an chromatin architecture modification gene and was found to be aberrantly expressed in LUAD.^30^ The oncogenic role of high mobility group A (HMGA) proteins stems from chromatin‐mediated activation of cancer‐driving genes such as E2F transcription factor 1 (E2F1), Activator protein‐1 (AP1), and Cyclin A1 (CCNA1), as well as the repression of tumor suppressive genes such as TP53.^31^ Moreover, the expression of noncoding genes, such as miRNAs, has been proved to be affected by the overexpression of HMGA proteins, leading to lung development through dysregulation of the cell cycle.^32^ In summary, these mRNAs are closely related to the regulation of inflammatory and hypoxia responses, regulation of artery smooth muscle cell proliferation and apoptosis, the response to the redox state, and the Bone morphogenetic protein (BMP) signaling pathway.

## CONCLUSION

In this study, we performed high‐throughput sequencing on specimens of patient tissues and carried out bioinformatics analysis on the sequencing data. We discovered several circRNAs that are abnormally expressed in LUAD tissues, and these circRNAs in turn contribute to carcinogenesis via interaction with miRNAs. Consequently, this study provides promising diagnostic markers and potential therapeutic targets, and lays the foundation for the future study of LUAD.

### ETHICAL APPROVAL AND CONSENT TO PARTICIPATE

This project was a retrospective study. Data were obtained by collecting tissue specimens and clinical pathological characteristics of patients who had undergone surgical resection. It did not require follow‐up of patients or direct contact with patients, and without personal privacy of patients. The collected data was de‐informatized and the subjects can no longer be found. The collected data was only used for statistical analysis and publication of papers, and did not involve personal privacy or commercial interests.

## CONSENT FOR PUBLICATION

All authors gave consent for the publication of manuscript.

## AUTHOR CONTRIBUTIONS

W.S. and Z.Y. conceived the project and designed the experiments. Y.C., X.H., X.H., Z.L., H.Z., B.L., J.H., and Y.X. performed the experiments. W.S., H.Y., and L.W. wrote the manuscript. Y.S., L.Z., D.Z., and Z.L. analyzed the data. J.H. supervised this work. All authors read and approved the final manuscript.

## CONFLICT OF INTEREST STATEMENT

The authors declare that they have no competing interests.

## Supporting information


**Table S1.** A total number of 5,241 circRNAs were detected in the specimens by second‐generation sequencing.

**Table S2.** Comparing to adjacent noncancerous tissues, LUAD tissues show upregulation of 4 of these circRNAs and downregulation of the other 14.

**Table S3.** A total of 217 target genes were screened.

**Table S4.** The results suggest that target genes are closely related to cell cycle, p53 signaling pathway, AMPK signaling pathway, and so on.

## Data Availability

All the data obtained and/or analyzed during the current study are available from the corresponding authors on reasonable request.
